# Desaturation index versus isotopically measured *de novo* lipogenesis as an indicator of acute systemic lipogenesis

**DOI:** 10.1186/s13104-015-1016-0

**Published:** 2015-02-23

**Authors:** Scott V Harding, Kevin P Bateman, Brian P Kennedy, Todd C Rideout, Peter JH Jones

**Affiliations:** Diabetes and Nutritional Sciences Division, King’s College London, 150 Stamford Street, London, SE1 9NH UK; Department of Pharmacokinetics, Pharmacodynamics and Drug Metabolism, Merck, One Merck Drive, Whitehouse Station, NJ 08889 USA; Kaneq Pharma, Montreal, QC Canada; Department of Exercise and Nutrition Sciences, University at Buffalo, Buffalo, NY 14214 USA; Richardson Centre for Functional Foods and Nutraceuticals, University of Manitoba, Winnipeg, R3T 6C5 MB Canada; Previously at Department of Biochemistry and Molecular Biology at the Merck Frosst Center for Therapeutic Research, Montreal, QC Canada

## Abstract

**Background:**

High carbohydrate feeding is known to increase plasma triglycerides as well as hepatic *de novo* lipogenesis (DNL) and may be implicated in the development of hepatic insulin resistance and fatty liver. Unfortunately, it is technically challenging to determine what proportion of circulating plasma triglycerides have been derived from the newly synthesized fatty acids in the postprandial period. The aims of this study were to 1) characterize the changes in the plasma postprandial total fatty acid pool in beagles following the consumption of meals containing 44% (Control) and 74% (High Sucrose) carbohydrate and 2) determine if changes in plasma fatty acid concentration and delta-9 desaturation index (DI) would be useful as simple and easy to measure biomarkers of systemic DNL.

**Findings:**

No differences in plasma total palmitic acid (16:0), stearic acid (18:0) and oleic acid (18:1) concentrations or delta-9 DI for the total 18:0 and 18:1 pools between High Sucrose and Controls were observed. However, newly synthesized 16:0 (2.6 ± 0.2% vs. 8.8 ± 2.0%; p = 0.016), 18:0 (0.93 ± 0.2% vs. 4.1 ± 1.7%; p = 0.007) and 18:1 (0.29 ± 0.09% vs. 3.5 ± 1.2%; p = 0.017) were higher in High Sucrose versus Control animals, respectively. Also, the delta-9 DI for the newly synthesized 18:0 and 18:1 pools was higher at 2 and 6 hours postprandial, with a pattern of change which supports the increased stearoyl-CoA desaturase (SCD-1) activity following high carbohydrate feeding followed by a down regulation of this enzyme.

**Conclusions:**

Our data show that high sucrose meals increase the relative contribution of systemic DNL produced fatty acids to the total postprandial plasma fatty acid pool. These data also show that a different pattern of both fatty acid synthesis and disposal occurs depending on energy and macronutrient profile of the meal. These changes are in spite of no observable changes in the plasma concentrations or ratios of the total fatty acid pool opposed to the observed changes in the newly synthesized fatty acid pool.

## Background

Diets high in carbohydrate are known to increase serum triglycerides (TG) [1-3] but the overall contribution of these diets to adiposity appears to be limited [4-6]. Overfeeding with simple carbohydrates to the point of exceeding total energy expenditure will yield higher absolute *de novo* lipogenesis (DNL) [7], but such situations are not normal over the long term in humans [8]. Therefore, carbohydrate induced DNL may not increase fat mass and adiposity to any great extent but the effect on hepatic lipid metabolism may be important in the development of hepatic insulin resistance and its related morbidities [9-11]. Hepatic insulin resistance may in fact be a protective mechanism which initiated by the liver during periods of excess energy substrate metabolism [12]. However, the contribution of simple carbohydrate to DNL during such periods invariably contributes to excess hepatic lipid production while rates of β-oxidation are generally reduced due to the increased malonyl-CoA concentrations produced via glycolysis and used as a substrate for DNL. Depending on the carbohydrate load consumed, the resulting DNL and TG production may exceed the VLDL secretion capacity resulting in increased liver fat content. Therefore, high carbohydrate diets – especially those high in simple sugars – are of particular interest the development of fatty liver diseases and hepatic insulin resistance.

Changes in hepatic lipid metabolism can be assessed by the changes in plasma lipid pools – namely the fatty acid and sterol pools. Because the main driver of the serum TG levels following high carbohydrate feeding tends to be DNL [13], changes in the relative proportion of newly synthesized fatty acids in the plasma are reflective of the DNL contribution to postprandial lipemia. Because of this direct relationship between dietary carbohydrate and the rate of postprandial DNL there is much interest in how this relationship contributes to the development of lipemia [14], obesity [2] and diabetes [15].

Overfeeding with both excess calories and simple carbohydrate is a very strong promoter of hepatic DNL [9-11] and understanding its impact on hepatic lipid metabolism and associated plasma biomarkers is important for understanding the development of hepatic insulin resistance. Therefore, the purpose of this study was to characterize the changes in the plasma postprandial total fatty acid pool in response to a high calorie simple carbohydrate meal. Our aim was to quantify plasma total fatty acid concentrations and hepatic fatty acid synthesis using isotopic techniques. We hypothesized that animals fed moderately excess calories and high sucrose would have higher plasma 16:0, 18:0 and 18:1 concentrations, higher desaturation indexes and proportion of newly synthesized fatty acids compared to controls. Our rationale for the examining the DI in relation to DNL is that while the terminal product of DNL is palmitate one of the preferred substrate in the synthesis of triglyceride is 18-carbon monounsaturated fatty acids [16]. Therefore, an increase in DNL may also alter the desaturation index of the plasma fatty acids proportional to rate of DNL and triglyceride synthesis. Ideally, the changes in fatty acid concentration and delta-9 DI would be then useful as cheap and easy to measure biomarkers of hepatic DNL if the patterns of change were confirmed by the isotopic data.

## Methods

### Experimental animals and diets

All procedures for *in vivo* experiments were approved by the Animal Care Committee at the Merck Frosst Centre for Therapeutic Research (Kirkland, Quebec, Canada) and were performed according to guidelines established by the Canadian Council on Animal Care [17]. Twelve adult male beagles from the Merck Frosst Centre for Therapeutic Research beagle colony, previously maintained on normal dog chow (Teklad Global Diet 2025, Harlan Teklad, Madison, WI), were randomized to receive a high sucrose meal (High Sucrose; Table 1) providing 3.9 kcal/g of diet with 74% of calories from carbohydrate or normal chow meal (Control) providing 3.5 kcal/g of diet with 42% of calories from carbohydrate (Table 2). Dogs receiving the high sucrose diet consumed a 400 g meal while those receiving the normal chow consumed a 250 g meal – 1560 kcal versus 875 kcal. Once meals were consumed each dog received 10 ml/kg body weight of deuterium oxide (D_2_O; 99.9% APE, CDN Isotopes, Pointe Claire, Quebec, Canada) administered by syringe. The syringes were then filled with 20 ml of distilled water and administered to the dog immediately after isotope dosing. Blood samples were taken, from a temporarily placed cannula, 1 hour before and 1, 2, 4, 6, 8 hours post D_2_O administration to measure the incorporation of deuterium into newly synthesized 16:0, 18:0 and 18:1. Only blood samples were collected for this experiment, there were no adverse events during the protocol and no animals were euthanized in this experiment.

**Table 1:** **High sucrose test meal composition**

	**Composition**	
**Ingredients**	**Amount (g)**	**Energy (Kcal)**
Casein	200	800
DL-Methionine	3	12
Corn starch, pre-gelatinized	48	192
Maltodextrin (Lodex 10)	75	300
Sucrose	750	3000
Cellulose	50	0
Soybean oil	50	450
Mineral mix S70003	50	0
Vitamin mix V90003	10	40
Choline Bitartrate	2	0
Total	1238	4794

**Table 2:** **Macronutrient composition of intervention and control meals**

**Macronutrient**	**High sucrose**		**Control** ^**1**^	
	**% composition (wt/wt)**	**% energy (% of total kcal)**	**% composition (wt/wt)**	**% energy (% of total kcal)**
Protein	16	17	26	32
Carbohydrate	71	74	61	42
Fat	4	9	11	26
Other	9	n/a	2	n/a
Macronutrient total	100	100	100	100
Energy density (kcal/g)	3.9		3.5	

^1^Commerical dog diet; Teklad Global 25% Protein Dog Diet-2025; Supplier: Harlan Teklad.

### Lipid extraction and fatty acid methyl ester conversion

Lipids were extracted and fatty acids converted to their methyl ester by standard procedures, previously reported [18]. Briefly, 0.5 ml of EDTA plasma and 125 μl of internal standard (heptadecanoic acid) were added to 8 ml of methanol followed by heating in 55°C water bath for 15 minutes. Hexane:chloroform (4:1, v/v; 24 ml) was then added followed by vortexing. Boron trifloride-methanol:hexane:methanol (7:6:7 v/v/v; 1 ml) was added to the dried lipid extract and fatty acid methyl esters were recovered following incubation at 100°C for 1 hour [18]. Samples were transferred to GC autosampler vials and stored at −80°C for analysis.

### Plasma fatty acid concentration and desaturation index

Fatty acid concentrations for palmitic, stearic and oleic acids were determined by gas chromatography with flame ionization (Agilent 6890 N, Mississauga, Canada). FAME were separated using a Supleco 2380 (30 m) with initial temperature of 50°C for 1 min then to 170°C (rate 40°C/min) then to 220°C (rate 8°C). Heptadecanoic acid was used as an internal standard and retention times were confirmed by using appropriate pure standards for each for each fatty acid. The Δ-9 desaturation index of the plasma fatty acids was calculated as the ratio of the total oleic acid pool to the total stearic acid pool [19]. Fatty acid concentrations were expressed as μg per ml of plasma.

### Fatty acid methyl ester deuterium incorporation

FAME deuterium incorporation was determined using GC separation on a Supleco 2380 column (30 m × 0.25 mm i.d. × 25 μm film thickness) followed by thermal conversion (pyrolysis) to hydrogen gas at 1450°C online and subsequent isotope ratio of deuterium:hydrogen measured via isotope ratio mass spectrometry (Delta V Plus, Thermo Electron Corporation, Massachusetts, USA). Deuterium enrichment was expressed as delta per mil (δ^2^H ‰) versus VSMOW was then converted to atom percent excess (APE).

### Statistical analysis

Plasma fatty acid concentrations, desaturation index and newly synthesized fatty acid concentrations were analyzed by multivariate analysis of variance where each values at each time point were treated as separate dependant variables and diet was designated as the independent variable. Data was analyzed using SPSS (version 21, IBM, Inc), 6/6 dogs per group used in statistical analysis and all values are expressed as means ± SEM.

## Findings

### Plasma fatty acid methyl ester concentration and desaturation index

**Figure 1:**
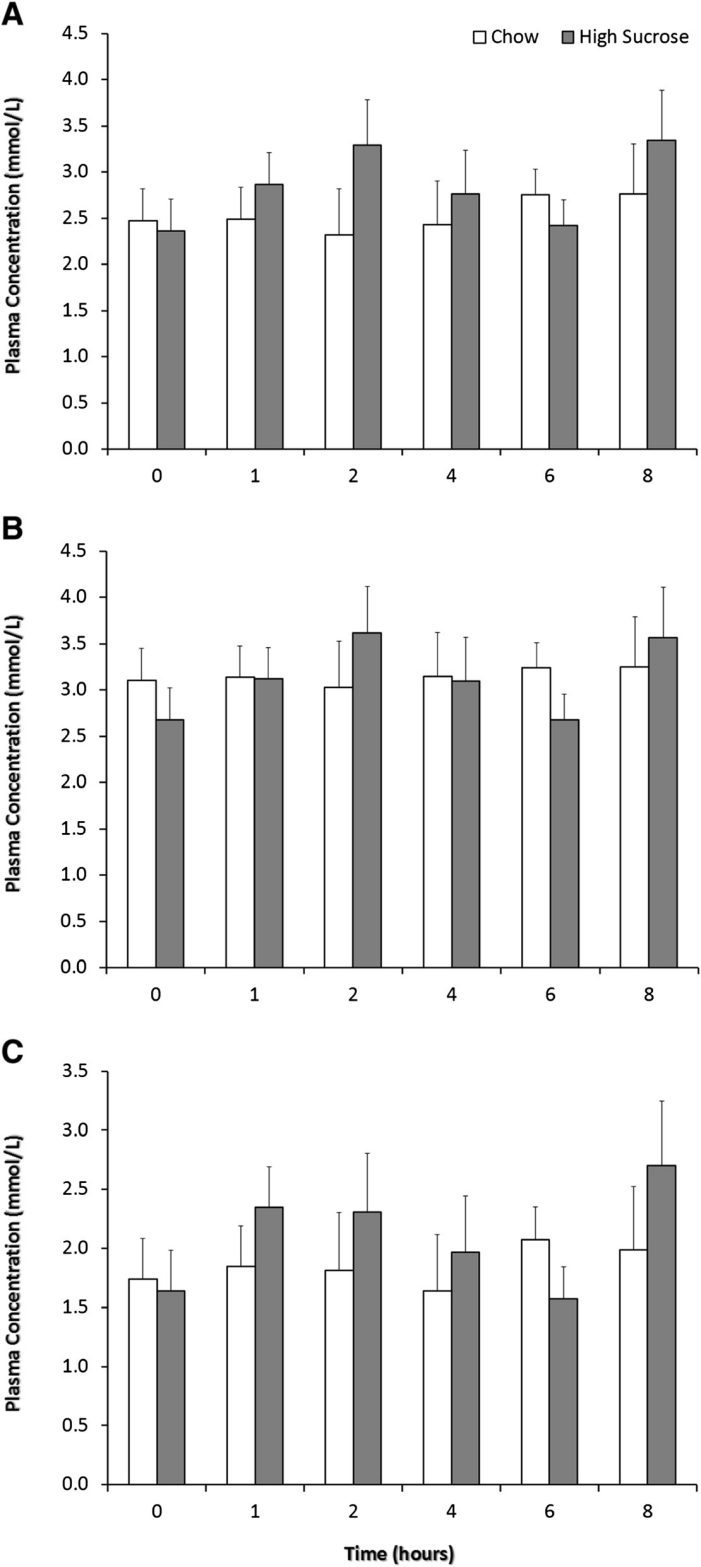
**The effect of high sucrose consumption on plasma fatty acid concentrations.** Beagles consuming either high sucrose meal providing 3.9 kcal/g of diet with 74% of calories from carbohydrate (High Sucrose; 400 g; 1560 kcal) or normal chow diet providing 3.5 kcal/g of diet with 44% of calories from carbohydrate (Control; 250 g; 875 kcal). **A)** Palmitic acid plasma concentrations, **B)** stearic acid plasma concentrations and **C)** oleic acid concentrations. Data are expressed as means ± SEM; n = 6.

**Figure 2:**
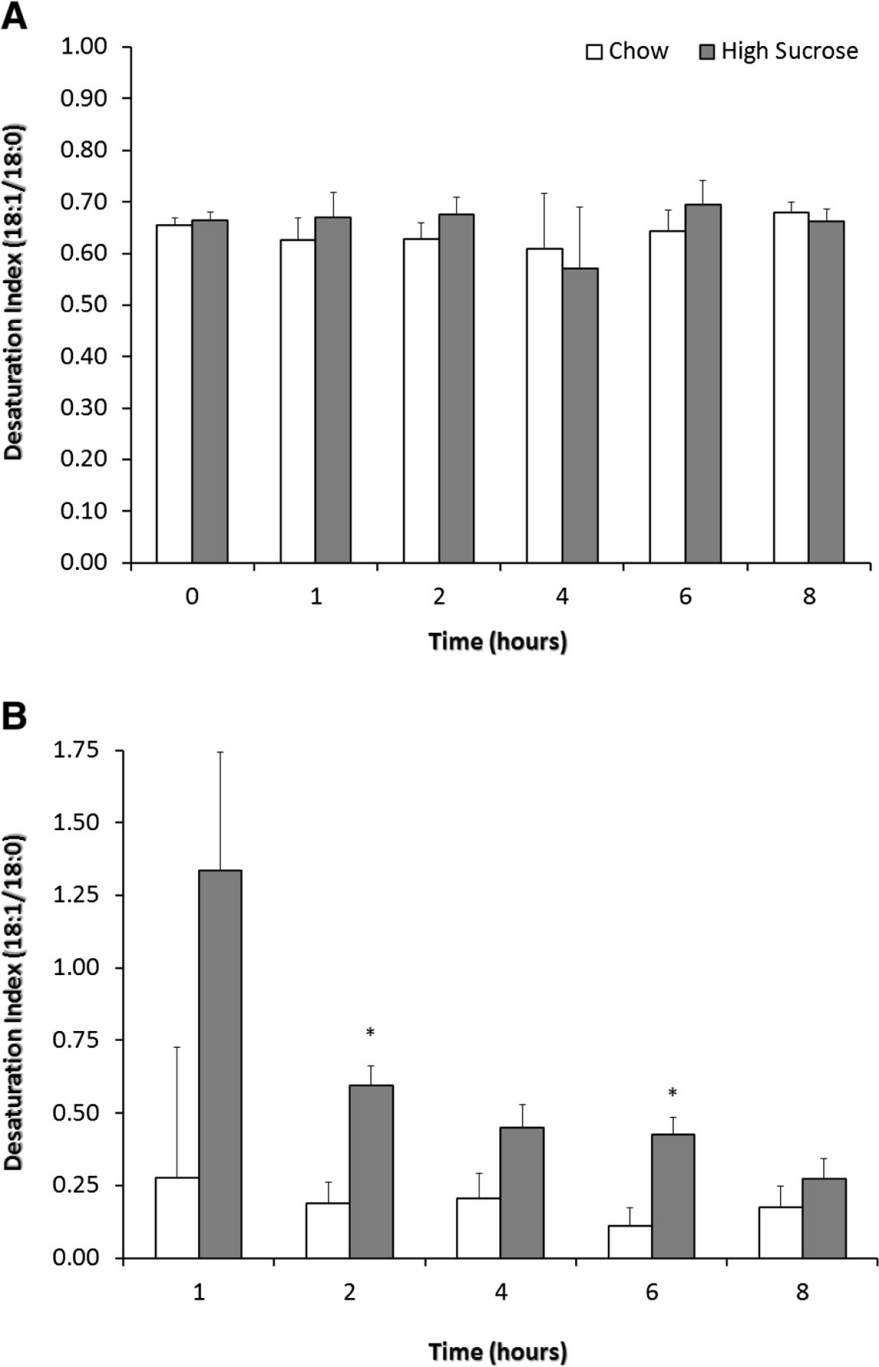
**The effect of high sucrose consumption on plasma Δ-9 desaturation index.** The delta-9 desaturation index is calculated as the ratio of the concentration of oleic acid (18:1) to stearic acid (18:0). **A)** The delta-9 desaturation index for total 18:1 and 18:0 pools from hours 0–8 of the postprandial period. **B)** The delta-9 desaturation index for newly synthesized 18:1 and 18:0 pools from hours 1–8 of the postprandial period. The pattern of change from a high desaturation index to low is indicative of the increased SCD-1 activity following a high carbohydrate meal. Data are expressed as means ± SEM; n = 6. * indicates p < 0.036 at each time point for High Sucrose versus Control.

Despite the overfeeding of sucrose there were no differences in postprandial plasma fatty acid concentrations for palmitic, stearic or oleic acids over the 8 hour post meal timeframe (Figure 1). Furthermore, the delta-9 DI did not differ for the total oleic and stearic acid pools (existing + dietary + newly synthesized) across the time points of the postprandial study period (Figure 2A) however the delta-9 DI for the newly synthesized fatty acids was different at hour 2 and hour 6 (Figure 2B). The pattern of change for the delta-9 DI of newly synthesized fatty acids was that of steadily decreasing.

### Newly synthesized plasma fatty acids

**Figure 3:**
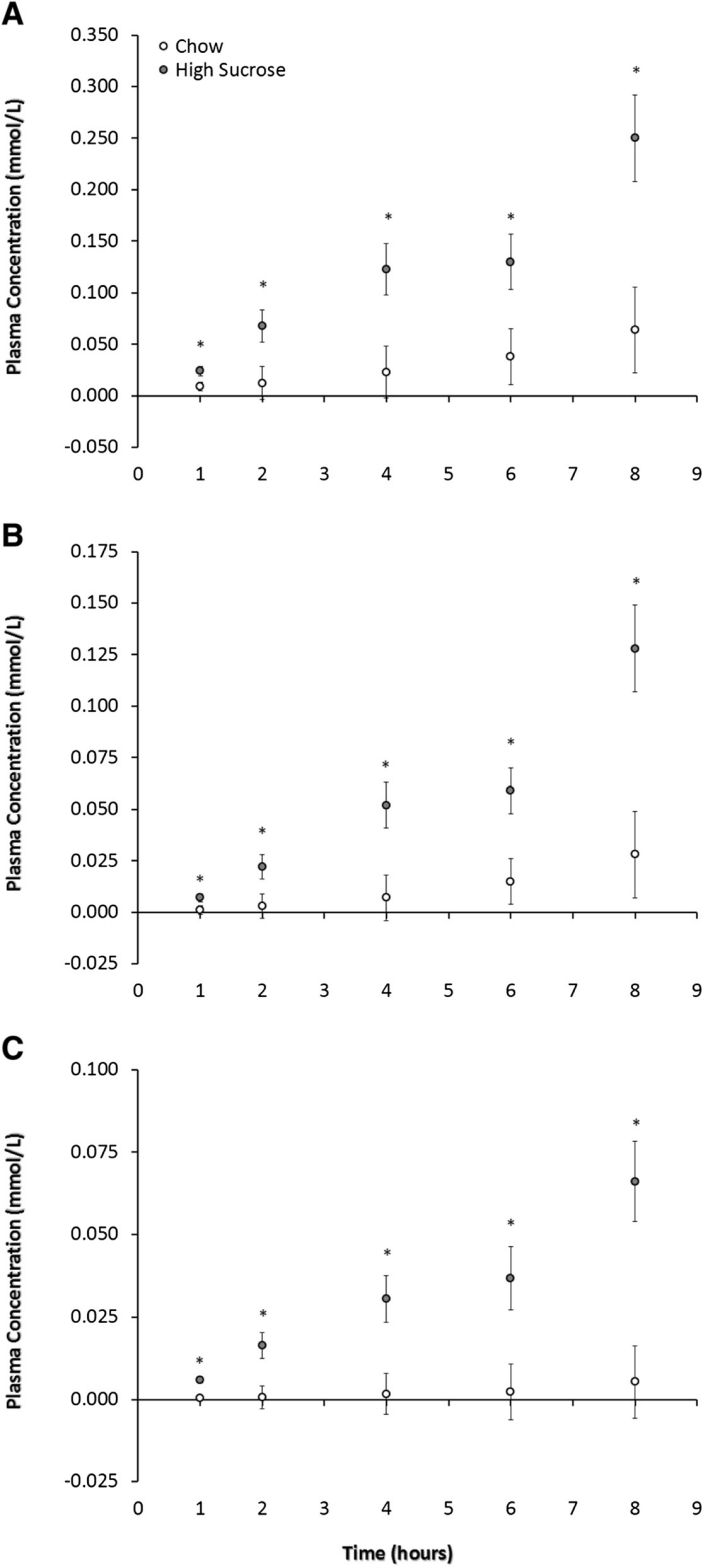
**The effect of high sucrose consumption on indicators of hepatic DNL.** Newly synthesized palmitic, stearic and oleic acid (expressed as concentration) at each time point in the postprandial period. **A)** Plasma newly synthesized palmitic acid, normalized to baseline natural enrichment. The proportion and absolute concentration of newly synthesized palmitic acid was higher at each time point measured in the postprandial period. **B)** Plasma newly synthesized stearic acid, normalized to baseline natural enrichment. The proportion and absolute concentration of newly synthesized stearic acid was higher at each time point measured in the postprandial period. **C)** Plasma newly synthesized oleic acid, normalized to baseline natural enrichment. The proportion and absolute concentration of newly synthesized oleic acid was higher at each time point measured in the postprandial period. Data are expressed as means ± SEM; n = 6. * indicates p < 0.036 at each time point for High Sucrose versus Control.

The amount of newly synthesized palmitic, stearic and oleic acid – expressed in terms of concentration – were higher following the high sucrose feeding versus control chow meal at each time point in the postprandial period (Figure 3A-3C). The total percent newly synthesized palmitic (2.6 ± 0.2% vs. 8.8 ± 2.0%; p = 0.016), stearic (0.93 ± 0.2% vs. 4.1 ± 1.7%; p = 0.007) and oleic (0.29 ± 0.09% vs. 3.5 ± 1.2%; p = 0.017) was also higher in the high sucrose fed animals, respectively.

## Discussion

Our objective in this study was to determine the effect of a high sucrose feeding on the production of liver derived newly synthesized fatty acids appearing in the plasma during the postprandial period and whether the delta-9 DI would serve as a useful biomarker of acute DNL. Our data show that dogs, previously unexposed to high sucrose diets, have significantly higher hepatic DNL as measured by newly synthesized fatty acids appearing in the plasma pool. In fact, dogs consuming the high sucrose had approximately 2.8-fold, 3.4-fold and 11-fold higher production of palmitic, stearic and oleic acid, respectively, over an 8 hour postprandial period. However, neither the plasma fatty acid concentration nor the delta-9 DI for the total stearic and oleic acid pools differed by treatment. However, the delta-9 DI for the newly synthesized stearic and oleic acid pools did differ at hour 2 and hour 6 of the postprandial period. This observation and the pattern of a decreasing delta-9 DI for the newly synthesized mirrors what would be expected based on an increased SCD-1 activity following a high carbohydrate meal [20].

A temporal pattern of postprandial DNL has been described by Timlin and Parks (2005) in humans which clearly have a steep departure from baseline TG concentrations and synthesis from an overnight fast that is compounded by meal frequency [4]. Concentrations of TG were not measured in this study and our plasma fatty acid concentrations fluctuated, but a pattern similar to that observed by Timlin and Parks for TG concentrations was not seen in these data over the 8 hours postprandial period [4]. Using the changes in the lipogenic fatty acid products may be useful biomarkers of hepatic DNL in response to high sucrose feeding because 1) higher levels of newly synthesized stearic acid indicates an increase in the elongation of palmitic acid by the hepatic fatty acid elongase-5 (elovl5) [21,22], and 2) the desaturation of stearic acid to oleic acid, via hepatic stearoyl-CoA desaturase 1 (SCD-1) [23] would also be increased. Unlike Timlin and Parks, we examined the contribution of total newly synthesized plasma fatty acids to the postprandial plasma fatty acid pool in order to simplify our analysis but saw no differences in the plasma palmitic, stearic and oleic acid concentrations and no differences in the delta-9 desaturation index.

As stated by Murphy [24], the use of deuterium incorporation and GC-IRMS is an excellent and sensitive technique to determine differences in fractional lipogenesis, but requires the determination of the specific fraction of deuterium which are introduced into the molecules understudy to precisely calculate absolute DNL. The model we present here demonstrates how the use of deuterium incorporation and GC-IRMS allow for the precise detection of label entry into the pools in question, even when those pools are extremely small. Also, the high sensitivity of IRMS and rapid distribution of ^2^H-label throughout total body water negates the need for long infusion to achieve isotopic steady state. Therefore, this isotopic technique not only provides a means to study differences in fractional DNL, but also the changes in DNL products through elongation and desaturation.

Two key limitations of this study are the multiple differences in the energy and composition of the meals and the inability to quantitate the contribution of all the tissues contributing to the total newly synthesized fatty acid pool (e.g. hepatic, adipose and intestinal). Firstly, the fatty acid composition, total carbohydrate load and total energy intake all exert an effect on the outcome measures presented here [25]. While this limits the ability to draw definitive conclusions as to the mechanisms responsible for the differing patterns of DNL. Similarly, the contribution of newly synthesized fatty acids by different tissues to the total fatty acid pool may indeed be directly affected by these dietary difference but there is no way to capture this information using the deuterium incorporation technique as used here. While the majority of the newly synthesized fatty acids are likely from hepatic DNL the consequences of having multiple difference in the meal compositions could impact the sources contributing to the total pool of newly synthesized fatty acids.

## Conclusion

In conclusion, our data illustrate that overfeeding with simple carbohydrates versus a normal basal diet in a single meal increases the relative contribution of total plasma fatty acids from DNL in the postprandial period. These data would also seem to indicate different patterns of both fatty acid synthesis and disposal in the short term following meals of high simple carbohydrate content. Unfortunately, the changes observed in the isotopic data were not observed in the fatty acid concentrations or the delta-9 DI. While the delta-9 DI of the total stearic and oleic acid pools did not prove to be a useful biomarker these data do illustrate the effectiveness of the deuterium incorporation technique for discriminating the very small changes in postprandial lipogenesis observed between treatments in this study and as a useful tool for potentially quantitating the activity of SCD-1 in the early postprandial period.

## Abbreviations

DNL: *de novo* lipogenesis
DI: Desaturation index
ELOVL5: Hepatic fatty acid elongase-5
FAME: Fatty acid methyl esters
GC-IRMS: Gas chromatography – isotope ratio mass spectrometry
SCD-1: Stearoyl-CoA desaturase 1
TG: Triglycerides
